# Predictors of complete remission of type 2 diabetes in patients over 65 years of age – a multicenter study

**DOI:** 10.1007/s11695-023-06705-0

**Published:** 2023-07-04

**Authors:** Natalia Dowgiałło-gornowicz, Paweł Jaworski, Maciej Walędziak, Paweł Lech, Alicja Kucharska, Piotr Major, Collaborative Study Group, Paula Franczak, Klaudia Juszczuk, Izabela Karpińska, Bartosz Katkowski, Grzegorz Kowalski, Michał Orłowski, Monika Proczko-Stepaniak, Michał Szymański, Mateusz Wityk

**Affiliations:** 1 Department of General, Minimally Invasive and Elderly Surgery, Collegium Medicum, University of Warmia and Mazury, 10-045 Olsztyn, Poland; 2 Department of General, Oncological and Digestive Tract Surgery, Centre of Postgraduate Medical Education, Orłowski Hospital, 00-416 Warsaw, Poland; 3 Department of General, Oncological, Metabolic and Thoracic Surgery, Military Institute of Medicine, 04-141 Warsaw, Poland; 4 Department of General Surgery, Pro-Medica Hospital, 19-300 Ełk, Poland; 5 2nd Department of General Surgery, Jagiellonian University Medical College, 30-688 Cracow, Poland; 6 Department of General and Oncological Surgery, Ceynowa Hospital, Wejherowo, Poland; 7 Department of General and Bariatric Surgery, Regional Specialist Hospital, Grudziądz, Poland; 8 Department of General and Vascular Surgery, Specialist Medical Center, Polanica Zdrój, Poland; 9 Surgery Clinic, Surgery Clinic Mazan, Katowice, Poland; 10 Department of General, Endocrine and Transplant Surgery, Medical University of Gdansk, Gdańsk, Poland; 11 Department of General and Oncological Surgery, Voivodeship Specialist Hospital, Słupsk, Poland

**Keywords:** Metabolic surgery, Bariatric surgery, Older patients, Elderly, Type 2 diabetes, Remission of obesity related diseases, Predictors

## Abstract

**Introduction:**

The type 2 diabetes (T2D) improvement rate in the elderly is high after bariatric and metabolic surgery, but not all patients achieve complete remission of disease. There are some predictors for T2D remission after bariatric surgery in patients of different ages, but there are few studies describing these factors in elderly patients. The study aimed to determine predictors for diabetes remission after bariatric surgery among patients over 65 years of age.

**Material and methods:**

A retrospective study analyzed patients over 65 years with T2D who underwent laparoscopic bariatric procedures in European country from 2008 to 2022. Multivariate logistic regression analysis was performed to obtain significant, independent risk factors.

**Results:**

The group consisted of 146 patients divided into two groups: responders (R) and nonresponders (NR). The complete remission of T2D was achieved in 51 (34.9%) patients. There were 95 (65.1%) patients in the NR group with partial remission, improvement, or no changes in T2D. The mean follow-up was 50.0 months. In a multivariate logistic regression analysis, a T2D duration of less than 5 years was found to be a predictor to T2D remission (OR = 5.5, *p* = 0.002), %EWL significantly corresponded to T2D remission (OR = 1.090, *p* = 0009).

**Conclusion:**

Bariatric and metabolic surgery appears to be a good option for T2D treatment in elderly patients. A shorter duration of T2D before surgery and higher %EWL after surgery were independent predictors of T2D remission in patients over 65 years of age.

**Graphical abstract:**

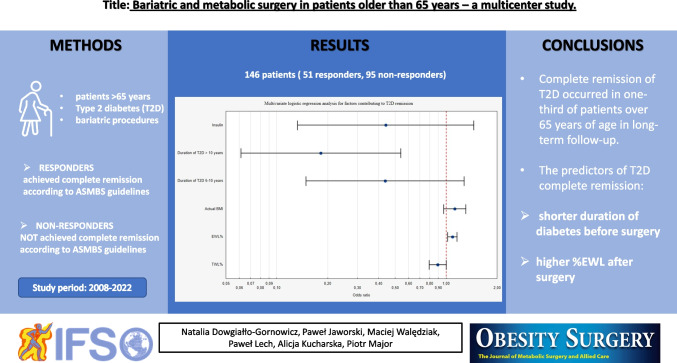

## Introduction

People are living longer, and the population is aging [1]. It is predicted that by 2030, 1 in 6 people in the world will be at least 60 years old. At that time, the share of the population aged 60 and over will increase by 40% for a decade. The rapidly increasing number of people also concerns patients with obesity. Obesity has reached epidemic proportions in the twenty-first century. Compared to 1975, the number of obese patients worldwide has tripled [2]. Overweight and obesity are major risk factors for many chronic diseases, including type 2 diabetes (T2D), hypertension, other cardiovascular diseases, and cancer [3]. Given the obesity pandemic and the aging population, we have increasingly elderly people with obesity worldwide who need treatment.

As the data of the World Health Organization show, there is no typical elderly person today. Some older people have similar physical and mental abilities as people half their age. Others experience a significant decline in fitness at a much younger age [2]. In the latest guidelines of the American Society for Metabolic and Bariatric Surgery regarding bariatric treatment in this age group, the approach to qualification for surgery in this age group has been slightly liberalized [4]. It was emphasized that not only age but also other factors play a significant role in eligibility for surgical treatment of obesity. Therefore, careful selection of patients is now recommended. Emphasizing the lack of support for age restriction in patient selection. The T2D improvement rate in the elderly is high after bariatric and metabolic surgery, but not all patients achieve complete remission of T2D [5, 6]. There are some predictors for T2D remission after bariatric surgery in patients of different ages, but there are few studies describing these factors in elderly patients [7–10].

### AIMS

The study aimed to determine predictors for diabetes remission after bariatric surgery among patients over 65 years of age.

## Materials and methods

**Fig. 1:**
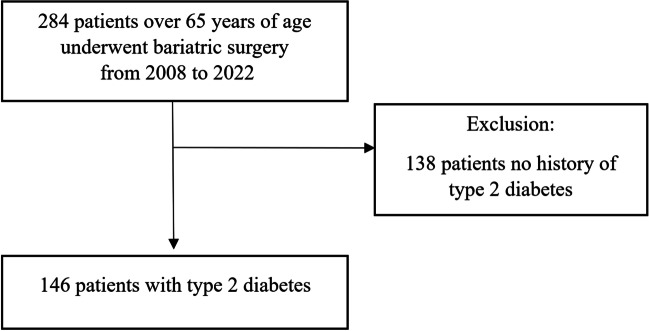
Flow chart of the study

It is a multicenter, retrospective analysis of a collected database of patients undergoing laparoscopic bariatric procedures in European country from 2008 to 2022. The data came from 11 bariatric centers. Each of these centers performs over 100 surgeries per year and is recommended by the National Society of Bariatric and Metabolic Surgery [11]. The whole group consisted of 284 patients. Inclusion criteria for this study were meeting the eligibility criteria for bariatric surgery, being over 65 years of age and suffering from T2D (Fig. 1). Patients with missing or inconsistent data were excluded from the study. The follow up rate is 78.0%. The analysis is in line with STROBE guidelines.

The study included 146 patients over the age of 65 suffering from T2D. The database contained demographic characteristics of patients (sex, age, maximal weight, weight before surgery, body mass index (BMI)) and information on T2D (type of treatment, duration of T2D). It also included information on the surgery (type of surgery, duration of surgery, length of hospital stay), and outcomes of bariatric treatment (current weight and BMI, percentage of excess weight loss (%EWL), percentage of total weight loss (%TWL), T2D remission). The outcomes of bariatric surgery were described in accordance with ASMBS outcome reporting [12]. Complete remission of T2D is a normal measure of glucose metabolism (HbA1c < 6%, fasting blood glucose (FBG) < 100 mg/dL) in the absence of antidiabetic medications. Partial remission is subdiabetic hyperglycemia (HbA1c 6%–6.4%, FBG 100–125 mg/dL) in the absence of antidiabetic medications. Improvement is statistically significant reduction in HbA1c and FBG not meeting criteria for remission or decrease in antidiabetic medications requirement. No remission is no significant changes in medication intake and laboratory test results [12]. Preoperative examinations were performed the day before surgery. Postoperative examinations are routinely performed at each follow-up visit annually after surgery. All results correspond to the follow-up time.

Surgical techniques and perioperative care protocols, including preoperative, intraoperative, and postoperative interventions, were standard at each participating center. Patients were treated by a multidisciplinary team of surgeons, physicians, nurses, nutritionists, and psychologists at each bariatric center.

### Statistical analysis

A descriptive statistical analysis was conducted. All data were analysed using Statistica software 13.PL (StatSoft Inc.). Continuous values were presented as the mean with standard deviation or medians with interquartile ranges when appropriate. Qualitative variables were compared using the Pearson χ-square test. Significant variables in univariate logistic regression models were then adjusted in multivariate analysis to obtain significant, independent risk factors and to calculate the OR with 95% confidence interval (CI). P values ≤ 0.05 were considered statistically significant.

### Ethical considerations

The data were completely anonymized. The study was conducted in accordance with the ethical standards of the 1964 Declaration of Helsinki and its subsequent amendments. The study was approved by the Bioethics Committee of the Military Chamber of Physicians in Poland (38/2023).

## Results

51 (34.9%) patients had complete T2D remission, 42 (28.8%) had partial T2D remission, 43 (29.5%) had improvement in T2D, and 10 (6.8%) had no changes in T2D. More than 90% of patients had at least an improvement in T2D. Patients were divided into two groups: responders (R) and nonresponders (NR). In group R, complete remission was achieved in 51 (34.9%) patients. There were 95 (65.1%) patients in the NR group with partial remission, improvement, or no changes in T2D. Table 1 compares the pre- and postsurgical characteristics of patients between the two groups. The mean follow-up time was 50.0 months (53.7 months for R group, 48.7 months for NR group).

**Table 1:** Characteristics of the patients. (IQR interquartile range, T2D type 2 diabetes, AGB adjustable gastric band, SG sleeve gastrectomy, OAGB one-anastomosis gastric bypass, RYGB Roux-en-Y gastric bypass, %TWL percentage of total weight loss, %EWL percentage of excess weight loss)

	Responders	Non-responders	p
N (%)	51 (34.9)	95 (65.1)	
Female/male, n (%)	35/16 (68.6)	54/41 (56.8)	0.164
Follow up, months (IQR)	53.7 (37.5–66.5)	48.4 (20.7–73.4)	0.359
Median age, years (IQR)	66 (66–68)	67 (66–68)	0.593
Median BMI, kg/m2 (IQR)	42.0 (37.1–47.9)	43.2 (40.2–46.2)	0.494
Median weight loss before surgery, kg (IQR)	5.0 (0–12)	4.0 (1–10)	0.792
Duration of T2D before surgery*			
< 5 years	24	19	0.002
5–10 years	12	20	
> 10 years	9	38	
Treatment of T2D before surgery			
Oral	44	67	0.034
Insulin	7	28	
Types of surgery			
AGB**	1	2	
SG	35	79	0.066
OAGB	9	6	
RYGB	6	8	
Operative time, min (IQR)	70.0 (55–90)	64.0 (55–90)	0.675
Length of stay, days (IQR)	2.0 (1–3)	2.0 (2–3)	0.015
Actual BMI, kg/m2 (IQR)	31.7 (27.8–36.1)	34.7 (32.0–37.9)	0.003
%TWL, % (IQR)	21.2 (15.8–32.8)	19.0 (12.9–25)	0.014
%EWL, % (IQR)	60.0 (41.5–83.0)	44.4 (30.6–57.4)	< 0.001

^*^data not available in all cases (88% R, 81% NR)

^**^due to a small sample number excluded from statistics

Patients from the R and NR groups did not differ statistically in terms of sex, age, BMI before surgery, weight loss before surgery, duration of follow up, type of surgery, or operative time. Statistically significant differences were observed in the duration and type of T2D treatment, and length of stay (*p* = 0.002, *p* = 0.034, *p* = 0.015, respectively). Among the outcomes, statistical significance was observed in correlation with actual BMI, %TWL% and %EWL (*p* = 0.003, *p* = 0.014, *p* < 0.001, respectively).

**Fig. 2:**
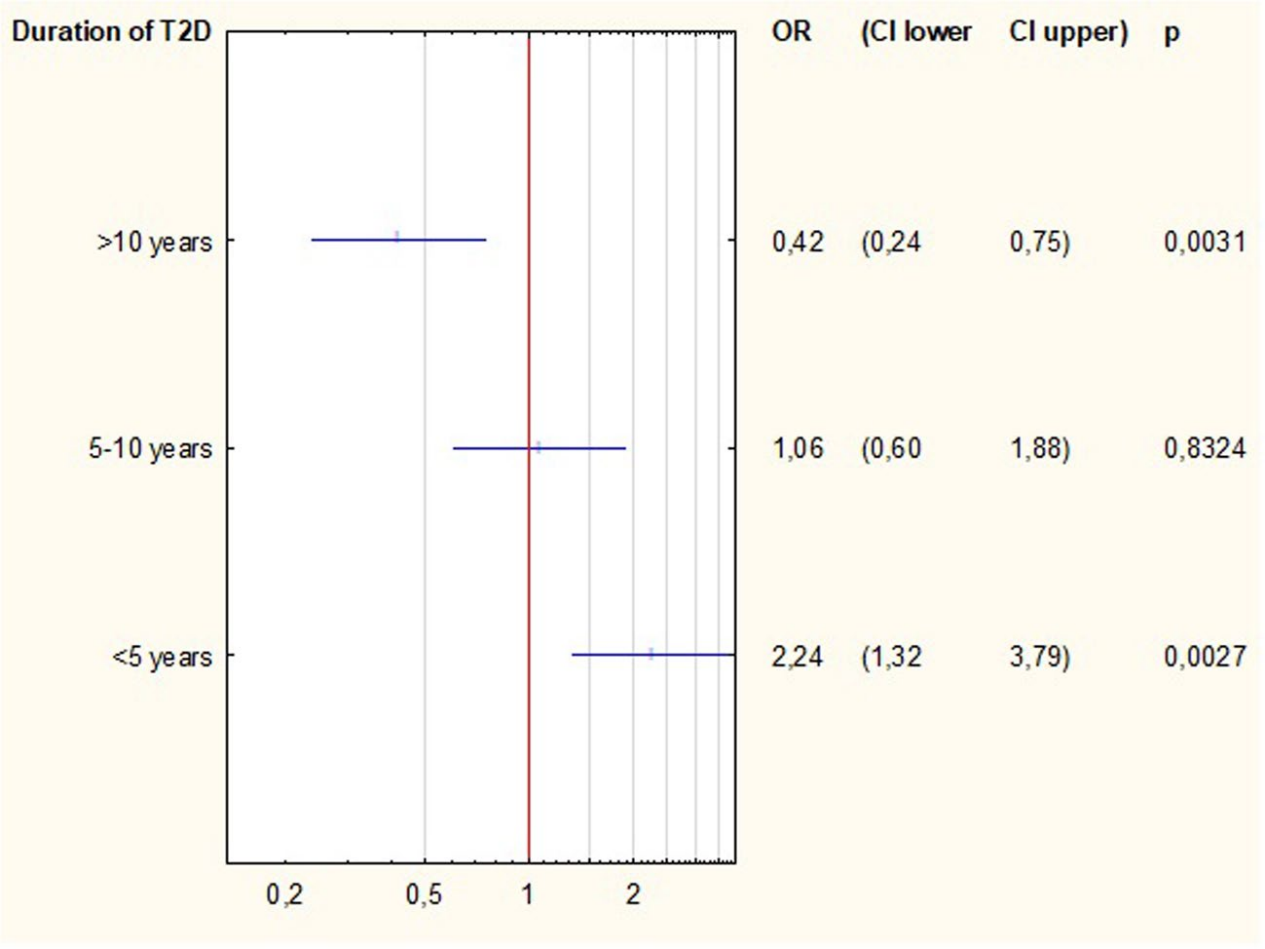
Univariate logistic regression for duration of type 2 diabetes. (OR odds ratio; CI confidence interval)

**Fig. 3:**
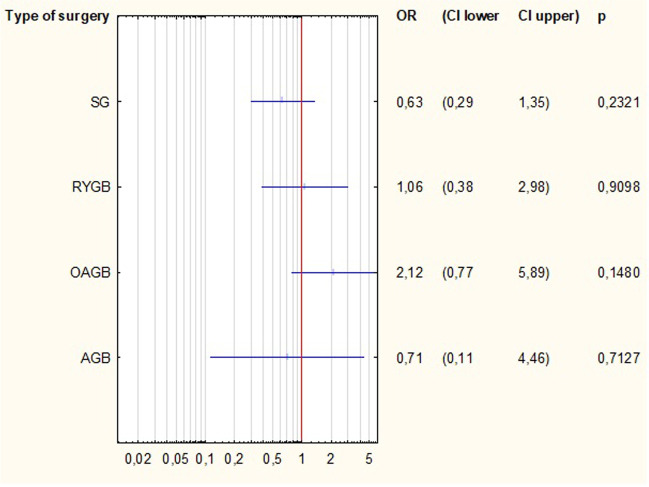
Univariate logistic regression for type of surgery. (OR odds ratio; CI confidence interval; SG sleeve gastrectomy; RYGB Roux-en-Y gastric bypass; OAGB one anastomosis gastric bypass; AGB adjusted gastric band)

All available factors contributing to the complete success of T2D treatment were analysed in univariate logistic regression models. Among the preoperative factors, type of treatment and duration of T2D significantly increased the odds ratio of complete T2D remission in patients over 65 years, Table 2. Preoperative oral medications and disease duration of less than 5 years were associated with a higher likelihood of complete remission of T2D (*p* = 0.0378, *p* = 0.003, respectively) (Fig. 2). Among the outcomes, actual BMI, %EWL and %TWL significantly increased the odds ratio of complete remission of T2D in patients over 65 years (*p* = 0.011, *p* < 0.001, *p* = 005, respectively), Table 2. There were no significant differences between the individual surgeries and their impact on the complete remission of T2D. However, OAGB had the greatest tendency to increase the probability of complete remission (OR = 2.12) (Fig. 3).

**Table 2:** Univariate logistic regression analysis for factors contributing to type 2 diabetes remission. (OR odds ratio; 95% CI 95% confidence interval, BMI body mass index, T2D type 2 diabetes, AGB adjustable gastric band, SG sleeve gastrectomy, OAGB one-anastomosis gastric bypass, RYGB Roux-en-Y gastric bypass, %TWL percentage of total weight loss, %EWL percentage of excess weight loss)

Variable		OR	95% CI	p
Median age		0.949	0.78–1.16	0.610
Median weight loss before surgery		0.988	0.94–1.03	0.594
Type of treatment				
	Oral	1.62	1.03–2.56	0.0378
	Insuline	0.62	0.39–0.97	0.0378
Length of T2D				
	< 5 years	2.24	1.32–3.79	0.003
	5–10 years	1.06	0.60–1.88	0.8324
	> 10 years	0.42	0.24–0.75	0.003
Type of surgery				
	AGB	0.71	0.11–4.46	0.712
	SG	0.63	0.29–1.35	0.232
	OAGB	2.12	0.77–5.89	0.148
	RYGB	1.06	0.38–2.98	0.910
Median length of stay		0.912	0.78–1.08	0.282
Median actual BMI		0.916	0.86–0.98	0.011
Median %EWL		1.026	1.01–1.04	< 0.001
Median %TWL		1.054	1.02–1.09	0.005

**Fig. 4:**
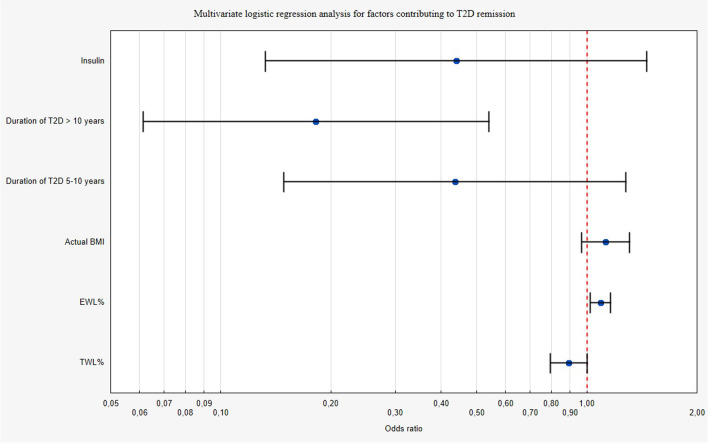
Multivariate logistic regression for factors contributing to type 2 diabetes remission. (BMI body mass index; %EWL percentage of excess weight loss; %TWL percentage of total weight loss)

All significant factors in univariate logistic regression were adjusted in multivariate analysis and presented in a forest plot (Fig. 4). In a multivariate logistic regression analysis, a T2D duration of less than 5 years was found to be a contributing factor to T2D remission (OR = 5.5, *p* = 0.002), Table 3. %EWL significantly corresponded to T2D remission (OR = 1.090, *p* = 0009), Table 3.

**Table 3:** Multivariate logistic regression analysis for factors contributing to type 2 diabetes remission. (OR odds ratio; 95% CI 95% confidence interval, BMI body mass index, T2D type 2 diabetes, %TWL percentage of total weight loss, %EWL percentage of excess weight loss)

Variable	OR	95% CI	p
Oral medicaments vs insulin	2.276	0.69–7.56	0.179
Duration of T2D < 5 years	5.50	1.86–16.32	0.002
Median actual BMI	1.126	0.97–1.31	0.126
Median EWL%	1.090	1.02–1.16	0.009
Median TWL%	0.893	0.80–1.00	0.056

There were 11 (7.5%) complications in the analyzed group. There were 6 (4.1%) 30-day Clavien Dindo III complications: 4 (2.7%) intraperitoneal bleedings, 1 (0.7%) leak, and 1 (0.7%) intraabdominal abscess. There were 2 (1.4%) tightening of sleeves requiring reoperation, 2 (1.4%) bile refluxes, and 1 (0.7%) mesenteric vein thrombosis. There were no postoperative deaths.

## Discussion

Our study is a retrospective analysis of 146 patients suffering from T2D over the age of 65 who underwent bariatric and metabolic surgery. To our knowledge, this is one of the few studies evaluating factors predisposing to T2D remission in patients over 65 years of age. It included data on one of the largest groups of patients over 65 years of age collected as a part of a multidisciplinary long-term follow-up reporting project.

Bariatric surgery achieves complete remission of T2D in significantly more patients than conventional therapy [13, 14]. This is associated not only with weight loss but also with metabolic changes taking place in the body [15, 16] Studies reported a nonsignificant comparison of older and younger patients in terms of improvement in obesity-related comorbidities [6, 17]. A recent systematic review by Giordano et al. showed that complete remission of T2D in elderly patients ranged from 33 to 83% [18]. The broad range results from considering both complete remission and partial remission, where a specific definition is not always given, as well as several types of procedures. In our study, complete remission of T2D was a normal measure of glucose metabolism in the absence of antidiabetic medications. Garofalo et al. reported a 34.8% complete remission of T2D after SG in patients over 65 years of age, which is similar to our results [19].

In recent studies we found that RYGB or OAGB compared to LSG is a predictor of diabetes remission [7, 13, 20]. Singh et al. in their meta-analysis showed that OAGB is not inferior to RYGB in terms of T2D remission [21]. The quoted analysis concerns patients of different ages. In our study of patients over 65 years of age, we found no significant differences between surgeries. However, OAGB tended to increase the likelihood of metabolic success of surgery.

In our study, a shorter duration of T2D was an independent predictor of T2D remission after bariatric surgery in patients over 65 years of age. Oral versus insulin use also increased this trend, but with no significant difference in multivariate logistic regression. Recent analyses showed the significance of both factors in patients of different ages [7, 9, 10]. This may be related to the functioning of pancreatic b-cells and insulin sensitivity [22, 23]. The longer the duration of T2D and the worse its control, the more difficult it is to achieve remission [22].

Moriconi et al. described that a shorter duration of T2D and better control of T2D were predictors for T2D remission after RYGB in 10-year follow-up [8]. On the other hand, weight loss had no effect on the long-term remission of T2D in patients of different ages [8]. Our study showed that %EWL in patients over 65 years of age could be a predictor of complete T2D remission.

Limitations of the study were its retrospective nature and SG's numerical superiority over other surgeries. Therefore, the comparison of the procedures may have not been sufficient and should be interpreted with caution. A small number of patients may have missed some variables that could lead to remission of T2D. Moreover, we do not have partial data on outcomes. The obtained results are the endpoint of the follow-up. However, due to the long follow-up time, the results seem to be relevant for bariatric and metabolic surgery.

## Conclusions

Complete remission of T2D occurred in one-third of patients over 65 years of age in long-term follow-up. Bariatric and metabolic surgery appears to be a good option for T2D treatment in elderly patients. A shorter duration of diabetes before surgery and higher %EWL after surgery were independent predictors of T2D remission in patients over 65 years of age.

## References

[1] WHO Fact sheet. Ageing and health. Accessed March 20, 2023

[2] WHO Fact sheet. Obesity and overweight. Accessed March 20, 2023

[3] Haslam DW, James WP (2005). Obesity. Lancet.

[4] Eisenberg D, Shikora SA, Aarts E, Aminian A, Angrisani L, Cohen RV (2022). American Society for Metabolic and Bariatric Surgery (ASMBS) and International Federation for the Surgery of Obesity and Metabolic Disorders (IFSO): Indications for Metabolic and Bariatric Surgery. Surg Obes Relat Dis.

[5] Prasad J, Vogels E, Dove JT, Wood C, Petrick AT, Parker DM (2019). Is age a real or perceived discriminator for bariatric surgery? A long-term analysis of bariatric surgery in the elderly. Surg Obes Relat Dis.

[6] Fernández-Ananín S, Ballester E, Gonzalo B, Codina C, Miñambres I, Pérez A (2022). Is Sleeve Gastrectomy as Effective in Older Patients as in Younger Patients? A Comparative Analysis of Weight Loss, Related Comorbidities, and Medication Requirements. Obes Surg.

[7] Dang JT, Sheppard C, Kim D, Switzer N, Shi X, Tian C, de Gara C, Karmali S, Birch DW (2019). Predictors for diabetes remission after bariatric surgery. Can J Surg.

[8] Moriconi D, Manca ML, Anselmino M, Rebelos E, Bellini R, Taddei S, Ferrannini E, Nannipieri M (2022). Predictors of type 2 diabetes relapse after Roux-en-Y Gastric Bypass: A ten-year follow-up study. Diabetes Metab..

[9] Varban OA, Bonham AJ, Carlin AM, Ghaferi AA, Finks JG, Ehlers AP (2022). Independent Predictors of Discontinuation of Diabetic Medication after Sleeve Gastrectomy and Gastric Bypass. J Am Coll Surg.

[10] Moradi M, Kabir A, Khalili D, Lakeh MM, Dodaran MS, Pazouki A, Kermansaravi M, Alibeigi P, Moazenzadeh H, Abdolhosseini MR, Eghbali F, Baradaran HR (2002). Type 2 diabetes remission after Roux-en-Y gastric bypass (RYGB), sleeve gastrectomy (SG), and one anastomosis gastric bypass (OAGB): results of the longitudinal assessment of bariatric surgery study. BMC Endocr Disord.

[11] Szeliga J, Wyleżoł M, Major P (2020). Metabolic and Bariatric Surgery Chapter of the Association of Polish Surgeons. Bariatric and metabolic surgery care standards. Videosurgery Other Miniinvasive Tech.

[12] Brethauer AS, Kim J, el Chaar M (2015). Standardized outcomes reporting in metabolic and bariatric surgery. Surg Obes Relat Dis.

[13] Schauer PR, Bhatt DL, Kirwan JP (2014). Bariatric surgery versus intensive medical therapy for diabetes — 3-year outcomes. N Engl J Med.

[14] Ikramuddin S, Korner J, Lee W-J (2013). Roux-en-Y gastric bypass vs intensive medical management for the control of type 2 diabetes, hypertension, and hyperlipidemia. JAMA.

[15] Mingrone G, Castagneto-Gissey L (2009). Mechanisms of early improvement/resolution of type 2 diabetes after bariatric surgery. Diabetes Metab.

[16] Mirghani H, Altedlawi AI (2023). Metabolic surgery versus usual care effects on diabetes remission: a systematic review and meta-analysis. Diabetol Metab Syndr.

[17] Bhandari M, Mathur W, Fobi M, Kosta S (2019). Outcomes of bariatric surgery in geriatric patients ≥ 65 years: single institution study. Obes Surg.

[18] Giordano S, Victorzon M (2015). Bariatric surgery in elderly patients: a systematic review. Clin Interv Aging.

[19] Garofalo F, Denis R, Pescarus R, Atlas H, Bacon SL, Garneau P (2017). Long-term outcome after laparoscopic sleeve gastrectomy in patients over 65 years old: a retrospective analysis. Surg Obes Relat Dis.

[20] Major P, Zarzycki P, Rymanowicz J (2022). Revisional operations among patients after surgical treatment of obesity: a multicenter Polish Revision Obesity Surgery Study (PROSS). Videosurgery Miniinv.

[21] Singh B, Saikaustubh Y, Singla V, Kumar A, Ahuja V, Gupta Y, Kashyap L, Aggarwal S (2023). One Anastomosis Gastric Bypass (OAGB) vs Roux en Y Gastric Bypass (RYGB) for Remission of T2DM in Patients with Morbid Obesity: a Randomized Controlled Trial. Obes Surg.

[22] Aminian A, Brethauer SA, Andalib A (2017). Individualized metabolic surgery score: procedure selection based on diabetes severity. Ann Surg.

[23] Liakh I, Proczko-Stepaniak M, Sledzinski M, Mika A (2022). Serum free fatty acid levels and insulin resistance in patients undergoing one-anastomosis gastric bypass. Wideochir Inne Tech Maloinwazyjne.

